# Performance-Boosted Interpretable ML via Optuna-SHAP: Uncovering Orientation-Driven Twinning in Mg Alloys

**DOI:** 10.3390/ma19122579

**Published:** 2026-06-15

**Authors:** Xuanyu Liu, Guoyao Chen, Xueting Wang, Pingli Mao, Ziqi Wei

**Affiliations:** 1 College of Interdisciplinary Sciences, Liaoning University of Technology, Jinzhou 121001, China; 2 Key Laboratory of Magnesium Alloys and the Processing Technology of Liaoning Province, Shenyang 110870, China; sauwangxt@163.com (X.W.);; 3 School of Materials Science and Engineering, Liaoning University of Technology, Jinzhou 121001, China; chen.guoyao@outlook.com; 4 School of Aerospace Engineering, Shenyang Aerospace University, Shenyang 110136, China; 5 School of Materials Science and Engineering, Shenyang University of Technology, Shenyang 110870, China

**Keywords:** magnesium alloys, twinning, machine learning, hyperparameter optimization, SHAP analysis, molecular dynamics

## Abstract

Machine learning (ML) is highly effective for modeling the complex factors governing twinning in magnesium (Mg) alloys, but it is often limited by challenges in hyperparameter optimization and a lack of interpretability, which reduce predictive accuracy and hinder mechanistic understanding. In this work, we present an enhanced interpretable ML framework that integrates Optuna for automated hyperparameter tuning using tree-structured Parzen estimators and SHapley Additive exPlanations (SHAP) for quantitative feature attribution. This approach delivers significant performance improvements, including F1-score gains of 6.33–11.84% on dataset T and AUC increases of up to 16.31% on dataset Y, outperforming previous benchmarks. When applied to a custom dataset derived from in situ EBSD tensile tests on Mg alloys and complemented by molecular dynamics (MD) simulations, SHAP analysis reveals a previously unrecognized grain shape-orientation effect: elongated grains with long-axis orientations of 20–80° relative to the tensile direction facilitate twinning nucleation, whereas orientations of 0–20° or 80–90° suppress it. Combined EBSD observations and MD simulations indicate that this effect arises from changes in boundary-segment orientation combinations, which regulate local constraint conditions, stress-transfer paths, and effective boundary resistance.

## 1. Introduction

Magnesium (Mg) alloys are promising lightweight structural materials owing to their low density and high specific strength [1,2,3,4]. However, their hexagonal close-packed (HCP) crystal structure restricts the number of available slip systems, rendering plastic deformation heavily reliant on twinning—particularly {10–12} tensile twinning and {10–11} compressive twinning [5,6]. Twinning plays a critical role in strain accommodation and ductility, yet it is governed by multiple interacting factors, including grain size, crystallographic orientation, and Schmid factor, which remain challenging to predict quantitatively [7,8,9,10].

Traditional experimental techniques, such as electron backscatter diffraction (EBSD) and transmission electron microscopy (TEM), have yielded valuable insights into twin nucleation and variant selection by resolving microstructural features at the grain scale [11,12,13]. In particular, in situ EBSD studies on AZ31 alloys—such as those by Wang et al. and Xu et al.—have directly monitored the formation, growth, and even detwinning of {10–12} tensile twins, showing that variants with high Schmid factors typically activate earlier and propagate faster, whereas deviations from Schmid’s law often stem from local strain accommodation by neighboring grains. Similarly, Zeng et al. [13] integrated in situ EBSD with mechanical modeling to elucidate the three-dimensional evolution of twinning in Mg alloys, further emphasizing the strong interplay among grain orientation, size, and local constraints.

Nevertheless, these methods are inherently labor-intensive and low-throughput, and their reliance on manual data interpretation hinders the establishment of robust quantitative correlations between microstructure and twinning probability [14,15,16]. Even recent high-throughput EBSD investigations, such as those by Fowler et al. [17] and Kasaeian et al. [18], still depend substantially on manual classification and expert judgment. Although automated high-throughput EBSD and in situ observation techniques have enhanced data acquisition efficiency [17,19], the analysis of large, multi-parameter datasets remains predominantly qualitative and expert-dependent. Consequently, comprehensively understanding the combined effects of grain size, orientation, and local stress state across thousands of grains continues to pose significant challenges [20,21]. There is therefore a growing need for computational methods capable of handling complex, nonlinear, and high-dimensional feature interactions to complement experimental observations and accelerate mechanistic discovery [22,23].

Machine learning (ML) has emerged as a powerful tool for analyzing such complex microstructural datasets [19,24,25,26,27,28,29,30,31]. For example, Tong et al. [19] evaluated five ML algorithms (decision trees, XGBoost, neural networks, support vector machines, and naïve Bayes) for twinning prediction in Mg alloys and identified grain diameter and c-axis orientation as dominant features. Yang et al. [24] employed Bayesian networks combined with feature ablation to investigate the role of neighboring grain rigidity in twin nucleation in AZ31 and Mg–1Al alloys. Similarly, Orme et al. [25,26] applied decision trees to EBSD data from AZ31 alloys, revealing key microstructural dependencies in twin activation. Peter et al. [27] further showed that integrating grain size, orientation, and strain parameters enables accurate prediction of twin nucleation in Mg–Y alloys. Despite these advances, existing ML approaches face two major limitations: (1) manual hyperparameter tuning is computationally costly and frequently yields suboptimal models [32,33,34]; and (2) conventional interpretability techniques, such as feature ablation or decision-tree visualization, fail to accurately quantify the contributions of correlated features, thereby restricting physical insights into twinning mechanisms [35,36].

To address these challenges, this study develops an integrated Optuna–SHAP framework for interpretable machine-learning analysis of twinning behavior in Mg alloys. The work is designed to answer three related questions. First, can automated hyperparameter optimization improve the predictive robustness of ML models across different Mg-alloy twinning datasets compared with previously reported benchmark models? Second, can SHAP-based explanations reproduce physically meaningful feature–twinning relationships in public datasets, thereby demonstrating that the optimized models capture relevant mechanisms rather than only statistical correlations? Third, when the validated framework is applied to a self-constructed in situ EBSD dataset, can it identify additional geometry-related descriptors of twinning behavior, and can these descriptors be further interpreted using representative EBSD observations and MD-based mechanical analysis? By combining public-dataset benchmarking, interpretable feature analysis, and experimental/simulation-based interpretation, this study aims to provide both an improved prediction framework and new insight into the role of grain shape orientation in Mg alloy twinning.

## 2. Method

### 2.1. Machine Learning Framework

The proposed framework integrates 18 mainstream machine learning algorithms, encompassing linear models, tree-based models, and ensemble methods such as AdaBoost and LightGBM [33,34]. To quantitatively assess feature contributions to twinning nucleation, model-specific SHAP interpreters—tailored for linear, tree-based, and other algorithms—are employed [37,38].

For hyperparameter optimization, a comprehensive search space is defined to cover key hyperparameters effectively. Optuna is then applied, using a hybrid strategy that first performs random grid search for broad exploration, followed by tree-structured Parzen estimator (TPE)-based refinement [36]. Each phase consists of 500 trials to maximize model performance. Robustness is evaluated via 10-fold cross-validation. In each cross-validation run, the dataset was divided into ten folds, with nine folds used for model training and the remaining fold used for evaluation. The model was reinitialized before each fold, and the held-out fold was not used during model fitting. The final performance was reported as the average score over the ten folds.

Performance is assessed using several metrics: macro-recall (the mean recall across the two classes), area under the receiver operating characteristic curve (ROC-AUC) for ranking performance, F1 score for balanced classification of twinned and untwinned grains, and overall accuracy. Final reported scores are averaged from the 10-fold cross-validation results. The framework is implemented in Python 3.12.12 using libraries including scikit-learn 1.8.0, XGBoost 3.2.0 [39], LightGBM 4.6.0, Optuna 4.8.0, and SHAP 0.51.0.

### 2.2. Experimental and Observation Methods

The starting material was a hot-extruded AZ31 (Mg–3Al–1Zn, wt%) magnesium alloy rod with a diameter of 45 mm. Dog-bone-shaped tensile specimens were machined from the rod using electrical discharge machining (EDM). EBSD data were acquired using a Gemini 300 scanning electron microscope (SEM, Carl Zeiss) equipped with an HKL Channel 5 EBSD system, employing a step size of 0.6 μm. Prior to EBSD characterization, the specimens were mechanically polished with silicon carbide (SiC) sandpaper and then subjected to argon ion etching for surface preparation.

**Figure 1:**
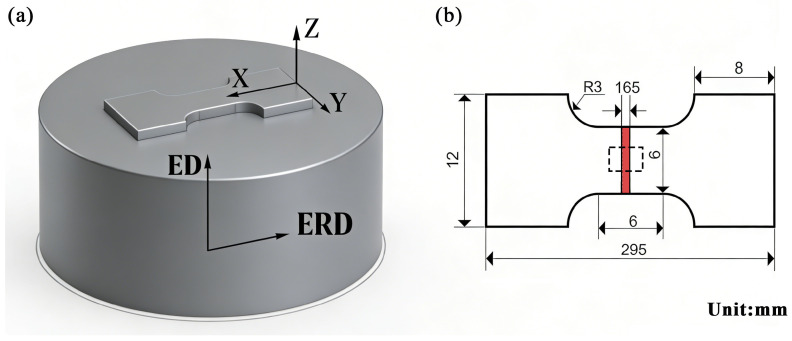
Schematic diagram of the specimen cutting area and observation area. (**a**) Schematic diagram of the cutting position of the dog-bone-shaped specimen in the magnesium alloy rod. (**b**) Schematic diagram of the in situ observation area of the dog-bone-shaped specimen.

**Figure 2:**
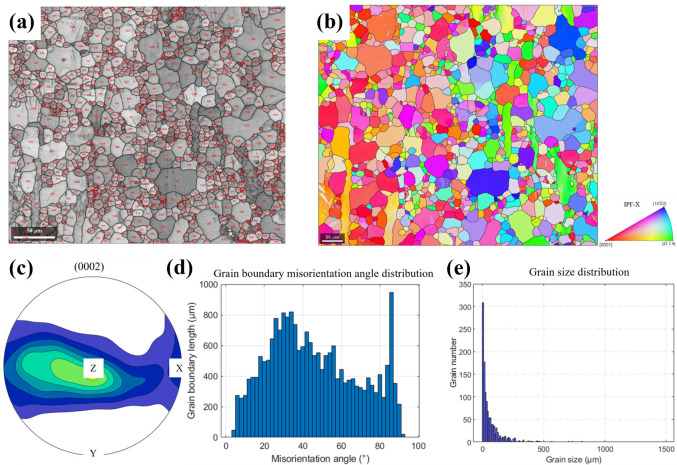
Microstructural characterization of the observed region prior to tensile deformation. (**a**) Band contrast (BC) map revealing grain morphology. (**b**) Inverse pole figure (IPF) map parallel to the extrusion direction (X-direction), showing crystallographic orientation distribution. (**c**) Orientation distribution function (ODF) plot illustrating the overall texture of the observed region. (**d**) Grain boundary misorientation angle distribution. (**e**) Grain size distribution histogram.

In situ tensile tests were conducted using a MICROTEST 2000E, (Gatan, Inc. (now a subsidiary of AMETEK, Inc. Pleasanton, United States of America) deformation stage (GATAN) mounted inside the SEM chamber, at a constant strain rate of 0.1 mm/min. The gauge section of the specimen was continuously monitored by EBSD during tensile deformation. The observed region and its microstructural characteristics before deformation are shown in Figure 1 and Figure 2.

### 2.3. Construction of the Dataset

This study utilizes two publicly available datasets (denoted as T and Y) from previous studies [19,24] and a self-constructed dataset (denoted as M) to evaluate the performance and interpretability of the proposed framework.

The public datasets T and Y were originally developed by Tong et al. and Yang et al., respectively. Detailed descriptions of their construction processes, including feature extraction and labeling strategies, can be found in the corresponding references. The primary differences between these datasets lie in the criteria for feature selection and the strain levels used to define twinning labels [19,24].

The self-constructed dataset M consists of 1011 individual grains extracted from the in situ EBSD maps of the extruded AZ31 alloy during tensile deformation. Candidate descriptors were first selected from physically meaningful categories, including grain size, crystallographic orientation, Schmid factor, and shape-related parameters. Shape-related descriptors were extracted from the reconstructed grain objects using the MTEX 6.0 toolbox in MATLAB r2025b. CaliperX and CaliperY represent the projected grain extents along the sample X and Y directions, respectively, while Diameter denotes the maximum Feret diameter of a grain, corresponding to the longest distance between two boundary points of the grain outline. In addition to these size-related descriptors, the long-axis angle (LAA) was introduced to describe grain shape orientation. LAA was defined as the angle between the major axis of the fitted ellipse of each grain and the tensile direction. Since the fitted long axis is non-directional, LAA was folded into the range of 0–90°, where 0° indicates that the grain long axis is approximately parallel to the tensile direction and 90° indicates that it is approximately perpendicular to the tensile direction. Feature selection was performed once on the full dataset before cross-validation. The retained feature set was then fixed and used consistently across all subsequent 10-fold cross-validation runs; no information from the held-out test folds was used during feature selection. Preliminary correlation analysis was used only as an exploratory tool to remove redundant or physically weak descriptors, and the retained feature set was fixed before cross-validation for all subsequent model evaluations.

For labeling twinning occurrence in dataset M, grains were classified as twinned (label 1) or untwinned (label 0) based on their state at 8% macroscopic strain. This strain level was selected because the fraction of twinned grains increases rapidly between 0% and 6% strain, then stabilizes at higher strains. Labeling at the stabilization stage (8% strain) better reflects the twinning behavior relevant to practical magnesium alloy processing, where materials with more stable twinning populations exhibit greater microstructural stability.

## 3. Result and Discussion

The Results and Discussion section is organized to progressively validate and apply the proposed Optuna–SHAP framework. Section 3.1 first evaluates the predictive performance of the optimized models on the two public datasets T and Y and the self-constructed dataset M, demonstrating the robustness of the framework across different data sources. Section 3.2 then examines the analytical capability of SHAP by comparing model-dependent feature attributions and verifying whether the framework can reproduce physically meaningful trends reported in previous studies. On this basis, Section 3.3 applies the validated framework to dataset M to identify a grain-shape-orientation effect associated with the long-axis angle (LAA). Section 3.4 and Section 3.5 further examine this framework-derived insight using representative in situ EBSD observations and molecular dynamics simulations, respectively.

### 3.1. Framework Performance Evaluation

**Figure 3:**
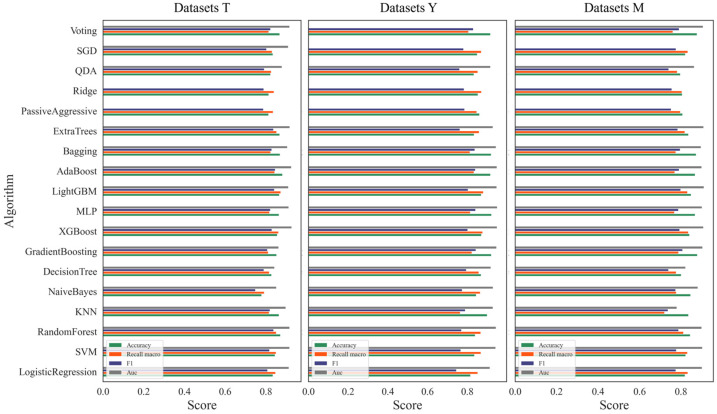
Performance metrics (accuracy, recall, F1 score, ROC AUC) for datasets T, Y, M.

The performance of the proposed framework was evaluated on the public datasets T and Y, as well as the custom dataset M, using the F1 score, area under the receiver operating characteristic curve (AUC), recall for the positive class (recall-1), and recall for the negative class (recall-0). The results are presented in Figure 3.

Because macro-recall was employed as the optimization objective—unlike the metrics used in prior studies on dataset T [19,24]—direct comparisons across all metrics may be influenced by these differing optimization conditions. Consequently, only F1 scores are compared for dataset T. Comparisons with the best previously reported models reveal that our optimized AdaBoost model achieved an F1 score of 0.84, outperforming the XGBoost benchmark of 0.79 (a 6.33% improvement). For the same algorithm type, the optimized support vector machine (SVM) improved its F1 score from 0.73 to 0.817 (an 11.84% increase).

For dataset Y, the optimized LightGBM model attained an AUC of 0.948, recall-1 of 0.89, and recall-0 of 0.865, surpassing the previous state-of-the-art Bayesian network model (AUC = 0.871, recall-1 = 0.879, recall-0 = 0.863) with relative improvements of 8.84%, 1.25%, and 0.23%, respectively. Even when comparing the same algorithm under non-optimized conditions in prior work, random forest exhibited substantial gains: AUC increased from 0.812 to 0.944 (16.31% improvement), recall-1 from 0.660 to 0.909 (37.77% improvement), and recall-0 decreased from 0.954 to 0.826 (−13.38%). This trade-off, inherent to macro-recall optimization, nevertheless reflects an overall enhancement in predictive performance.

On the custom dataset M, all optimized models demonstrated consistently strong and comparable performance, further underscoring the robustness and effectiveness of the framework across diverse datasets.

These results demonstrate that the Optuna-optimized models provide reliable predictive performance across datasets with different feature definitions and labeling strategies. However, high predictive accuracy alone does not guarantee that the model captures physically meaningful mechanisms. Therefore, before applying the framework to the self-constructed dataset M for mechanistic discovery, we next evaluate whether SHAP-based explanations can reproduce and extend known feature–twinning relationships in the public datasets.

### 3.2. Framework Analysis Capability Evaluation

After confirming the predictive performance of the optimized models, we further evaluated whether the SHAPs generated by the framework were physically meaningful. This section therefore focuses not on model accuracy, but on whether different algorithms provide consistent and interpretable feature–twinning relationships on public datasets T and Y.

**Figure 4:**
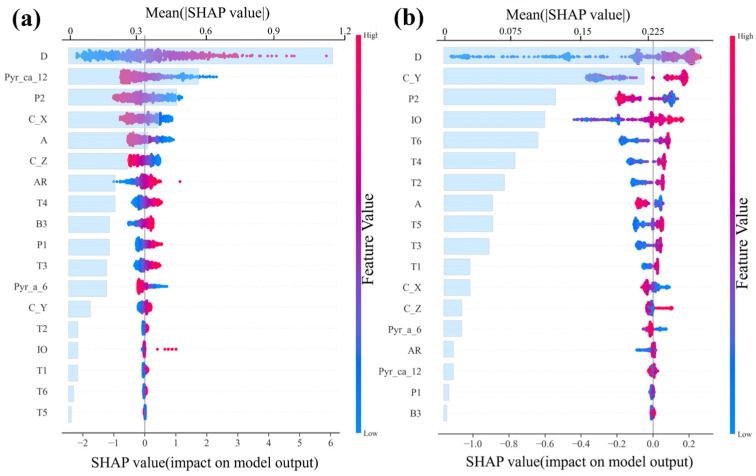
SHAP summary and feature importance plots for dataset T: (**a**) LR; (**b**) XGBoost.

Different machine learning algorithms exhibit varying sensitivities to feature types (e.g., linear versus nonlinear), which can influence subsequent interpretability analyses. To evaluate this effect, SHAP summary and feature importance plots were generated using logistic regression (LR) and XGBoost 3.2.0 on dataset T (Figure 4a,b). The results show variations in feature importance rankings between the two models, consistent with prior studies and confirming that algorithm choice affects feature perception.

**Figure 5:**
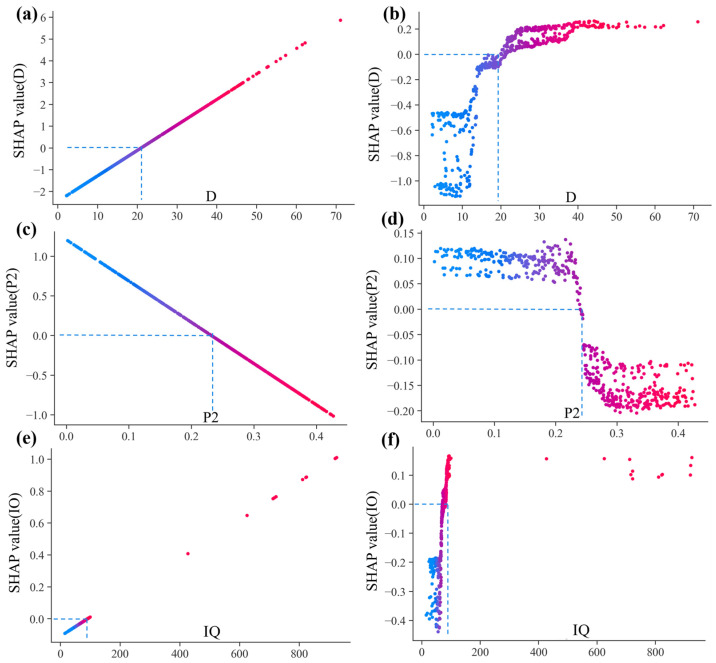
SHAP dependence plots for dataset T: (**a**,**c**,**e**) LR for features D, P2, and IQ; (**b**,**d**,**f**) XGBoost for the corresponding features. The color ranging from blue to red indicates that the SHAP values of the points increase from low to high.

To investigate whether algorithm-specific feature weighting affects the inferred physical mechanisms, SHAP dependence plots were examined for three key features in dataset T: grain size (D), secondary prismatic slip Schmid factor (P2), and EBSD image quality (IQ). Logistic regression (LR) and XGBoost were compared, with the LR results shown on the left and the XGBoost results on the right in Figure 5a–f.

The analysis indicates that, although the rate of change in twinning influence varies between models for the same feature, both the LR and XGBoost models show similar trends in terms of positive and negative SHAP contributions. For example, as shown in Figure 5a,b, the grain size (D) transition point is approximately 21 μm (derived from labels at 4% strain; this value may vary with strain level [12,32]). Figure 5c,d reveal a transition point of 0.23 for the secondary prismatic slip Schmid factor (P2), above which twinning is suppressed and below which it is promoted. Similarly, Figure 5e,f show a transition point of 100 for EBSD image quality (IQ), with higher values favoring twinning nucleation and lower values inhibiting it.

**Figure 6:**
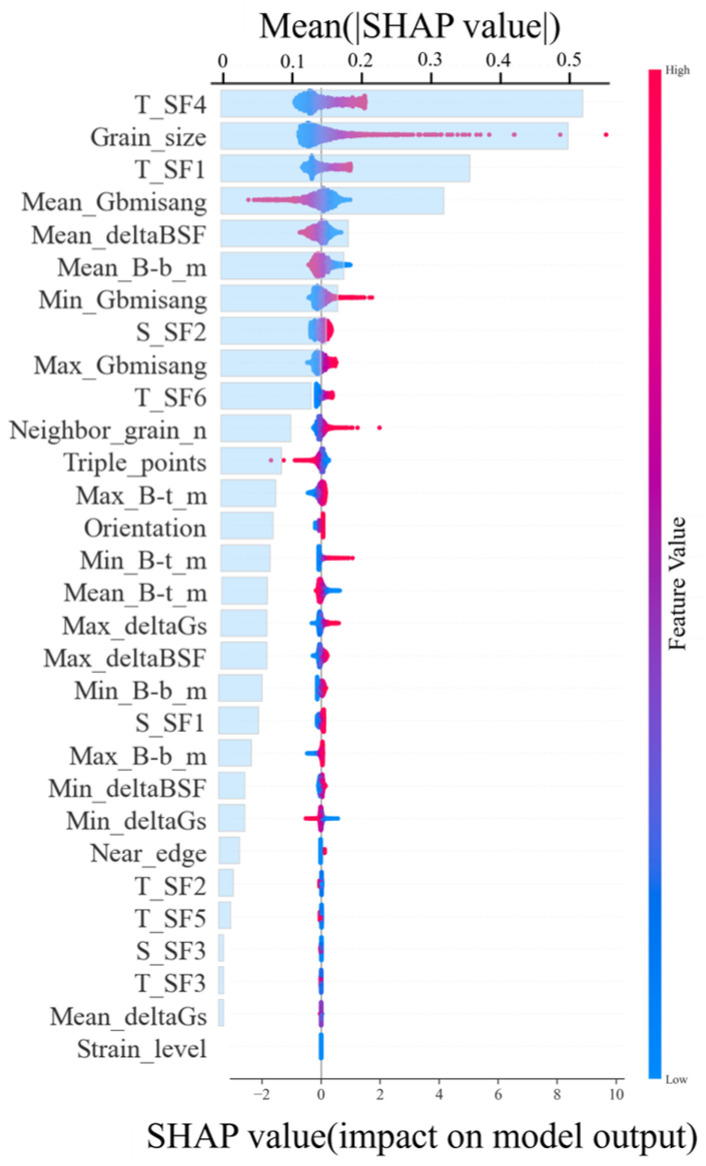
SHAP summary and feature importance plots for dataset Y obtained using the LR model.

To further assess the analytical capabilities of the framework, SHAP analysis was performed using the LR model on dataset Y. The resulting SHAP summary and feature importance plots are shown in Figure 6. These results reveal the relative weight of different features and the distribution of SHAP values across individual grains, which are consistent with findings from previous studies.

**Figure 7:**
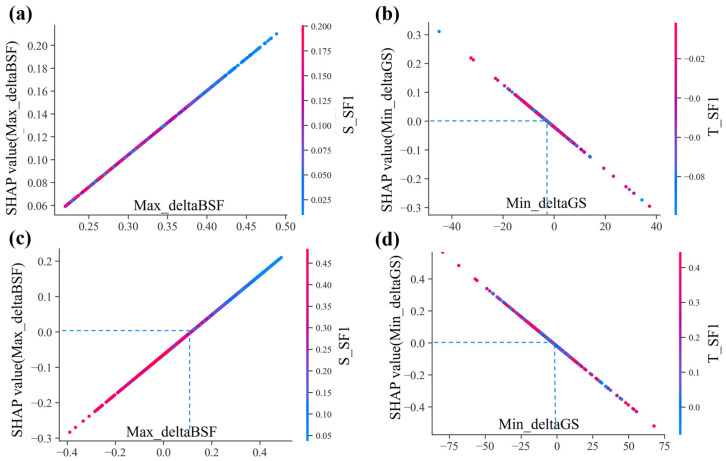
SHAP analysis for Datasets Y: (**a**) Max_deltaBSF and S_SF1 for grains <7 µm with T_SF1 > 0.46, (**b**) Min_deltaGS and T_SF1 for grains with T_SF1 < 0, (**c**) Max_deltaBSF and S_SF1 for all grains, (**d**) Min_deltaGS and T_SF1 for all grains.

Previous studies employing feature ablation methods identified that grains with a maximum basal slip Schmid factor difference (Max_deltaBSF) > 0.22, a primary slip Schmid factor (S_SF1) < 0.22, a high twinning Schmid factor (T_SF1 > 0.46), and a grain size < 7 μm are more prone to twinning. Figure 7a presents the SHAP dependence plot for grains satisfying Max_deltaBSF > 0.22 and S_SF1 < 0.22, validating these findings. Additionally, Figure 7c displays the SHAP dependence plot for all grains, revealing that lower Max_deltaBSF values also suppress twinning. Similarly, Figure 7b,d indicate that the minimum grain size difference (Min_deltaGS) has a limited correlation with T_SF1 in influencing twinning across all grains. These findings build upon and advance prior work.

Overall, the analyses on datasets T and Y show that the proposed framework not only improves prediction accuracy but also provides physically consistent explanations that agree with previously reported mechanisms and reveal additional feature dependencies. Having validated both predictive performance and interpretability on public datasets, we then applied the framework to the self-constructed dataset M to explore whether it could uncover new twinning-related factors.

### 3.3. Framework Application and Key Findings

#### 3.3.1. Identification of LAA as a Stable Secondary Feature

**Figure 8:**
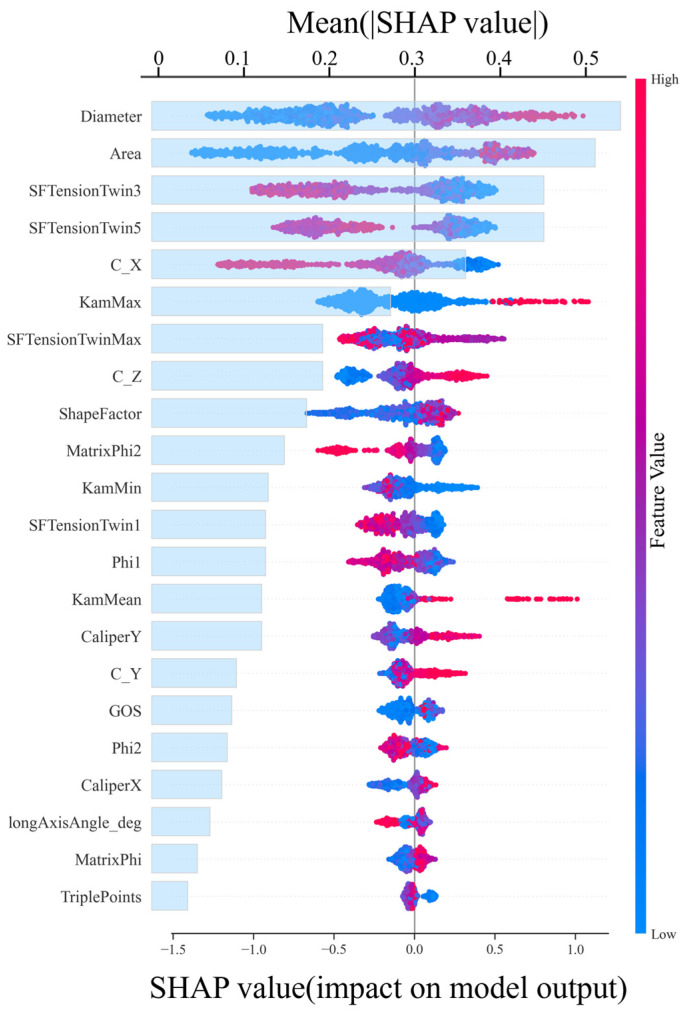
SHAP summary and feature importance plots for dataset M.

To further investigate the framework’s capability to uncover novel phenomena, we applied SHAP analysis to the self-constructed dataset M. XGBoost was selected for the subsequent interpretation because the preceding analysis on dataset T showed that both LR and XGBoost produced consistent SHAP-based transition trends for key features, indicating that the inferred physical tendencies were not strongly dependent on the algorithm type. In addition, XGBoost achieved among the best predictive performance on dataset M and provides efficient TreeSHAP implementation, making it suitable for analyzing nonlinear feature effects, feature interactions, and main-effect trends in the custom dataset. Figure 8 presents the SHAP summary plot and feature importance ranking obtained from the optimized XGBoost model. It can be seen that LAA does not rank among the most important features, suggesting that it may not be a dominant factor controlling twinning behavior. Nevertheless, the corresponding SHAP values still exhibit a noticeable distribution range, indicating that this feature makes a stable but non-dominant statistical contribution to the model output. In other words, although the overall geometrical elongation direction of a grain may not be the primary factor controlling twin nucleation, the shape-orientation information captured by LAA still exerts a detectable influence on the predicted twinning probability.

Based on this result, two further questions need to be addressed. First, does the shape-orientation effect represented by LAA merely arise from its correlation with the projected grain size along the tensile direction, that is, is LAA simply another expression of CaliperX? Second, if LAA is not a simple surrogate for CaliperX, does it exhibit an identifiable main-effect trend associated with twinning occurrence? Therefore, we first analyze the correlations and interaction relationships between LAA, CaliperX, and other shape-related descriptors. We then further examine the statistical contribution of LAA using a conditional subsample in which the collinearity between LAA and CaliperX is reduced. Finally, the potential physical meaning of LAA is discussed based on its SHAP main-effect trend.

#### 3.3.2. Excluding the CaliperX Projection Effect

**Figure 9:**
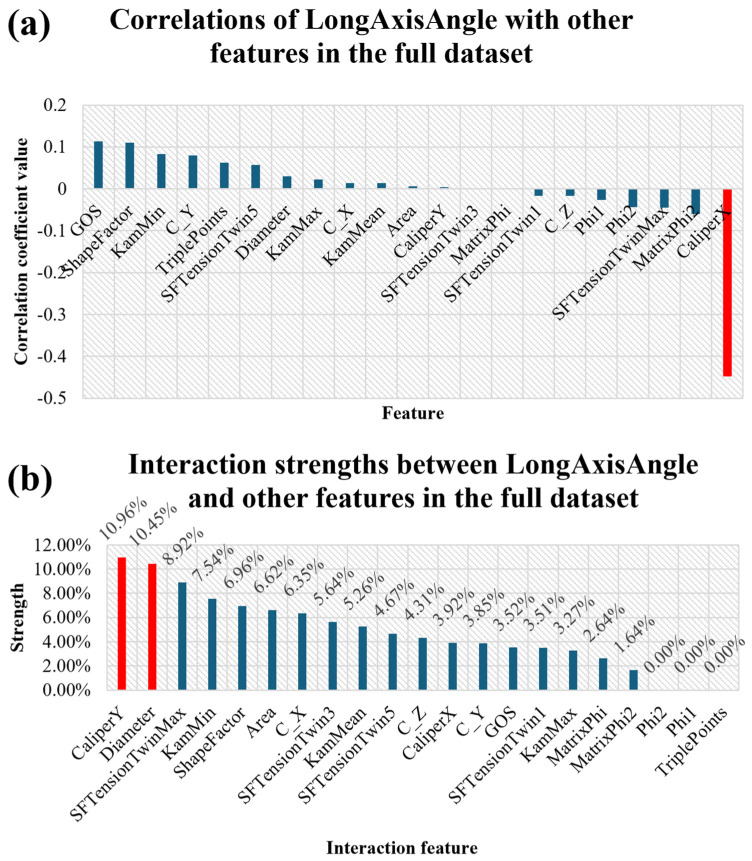
Correlation and interaction-strength rankings between LAA and other features before multicollinearity reduction: (**a**) Pearson correlation coefficients between LAA and all other features; (**b**) interaction strengths between LAA and all other features.

Before filtering, LAA showed a certain degree of correlation with CaliperX, as shown in Figure 9a, and exhibited strong interactions with CaliperY and Diameter, as shown in Figure 9b.

**Figure 10:**
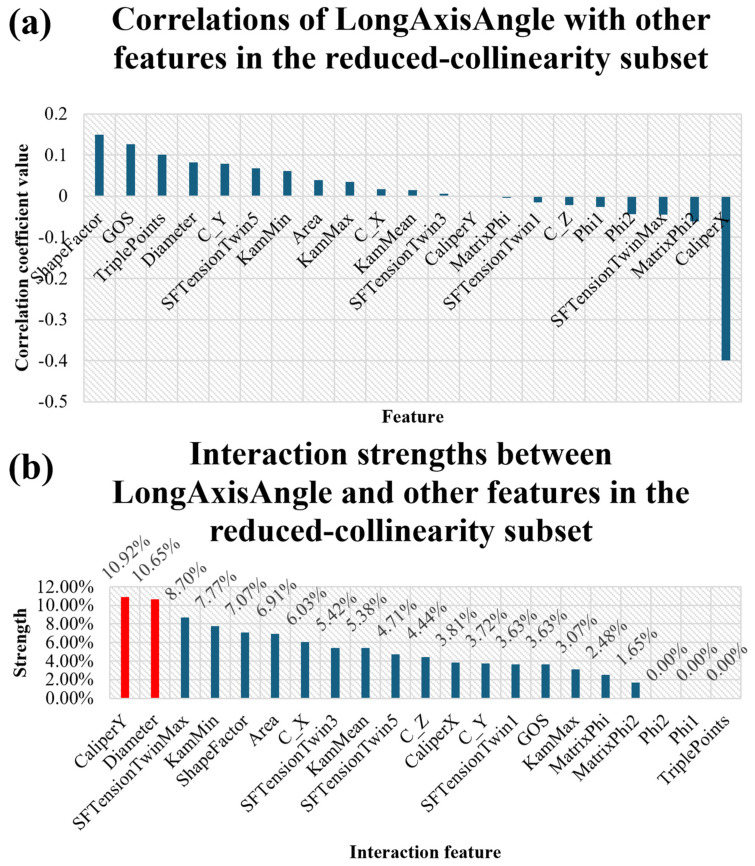
Correlation and interaction-strength rankings between LAA and other features after multicollinearity reduction: (**a**) Pearson correlation coefficients between LAA and all other features; (**b**) interaction strengths between LAA and all other features.

To minimize the possible interference of CaliperX in the interpretation of LAA, a conditional subsample was constructed using univariate linear regression and residual-based filtering. The subsample was selected such that the absolute Pearson correlation coefficient between LAA and CaliperX was lower than 0.4. The correlation, distribution, and interaction behavior of LAA were then re-evaluated within this subsample. As shown in Figure 10a, after filtering, no feature exhibited an absolute correlation coefficient greater than 0.4 with LAA, indicating that the collinearity between LAA and CaliperX had been substantially weakened. Moreover, Figure 10b shows that, even under this condition, CaliperY and Diameter remained the two features with the strongest interactions with LAA, and their interaction strengths were still more than twice the average level. These results indicate that the role of LAA should not be simply interpreted as a projected-size alternative to CaliperX. After reducing the influence of collinearity, LAA still participates in twinning prediction as a relatively independent descriptor of grain shape orientation, and its statistical contribution does not disappear.

In summary, although LAA shows a certain degree of correlation with CaliperX, it still maintains strong interactions with CaliperY and Diameter after the collinearity between LAA and CaliperX is reduced. This indicates that LAA is not simply an alternative representation of CaliperX but can provide additional information for twinning prediction beyond the intrinsic grain-size descriptors. More specifically, LAA does not merely reflect the grain dimension along a particular direction; rather, it is more likely associated with the overall grain-shape orientation and its statistical relationship with the tensile direction. How this feature further affects local constraint conditions, stress-transfer behavior, and twin nucleation still requires further analysis based on the main-effect interval characteristics of LAA and representative grain-scale observations discussed in the following sections.

#### 3.3.3. Main-Effect Behavior and Physical Interpretation of LAA

**Figure 11:**
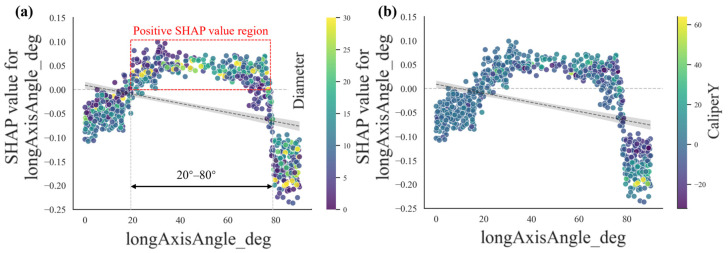
SHAP dependence plots of LAA for two highly interacting features after multicollinearity reduction: (**a**) Diameter; (**b**) CaliperY.

The previous subsection demonstrated that LAA is not simply an alternative projection-based representation of CaliperX but provides additional information beyond purely size-related descriptors. On this basis, this subsection further examines the main-effect behavior of LAA on twinning propensity and whether this effect exhibits a threshold-like partitioning trend. To this end, Figure 11 presents the SHAP dependence plots of LAA after reducing its collinearity with CaliperX, with CaliperY and Diameter used as the interacting features. Although locally clustered distributions can be observed for data points with different colors, substantial overlap remains among different colored data points within the same LAA range. No clear regime-dependent relationship dominated by a single size descriptor is observed. This indicates that the modulation of the LAA effect by CaliperY and Diameter is more likely to be continuous and dispersed, rather than a discrete conditional mechanism that can be characterized by a single critical value. In other words, intrinsic grain-size-related descriptors can affect the strength of the LAA effect on twinning, but this influence does not exhibit an obvious “threshold-switching” behavior.

Further examination of the SHAP-value distribution in Figure 11 shows that the effect of LAA on twinning generally follows a statistical main-effect trend characterized by promotion in the intermediate range and suppression near both ends. When LAA falls within approximately 20–80°, the corresponding SHAP values are generally higher, indicating that grains in this range are more prone to twinning. In contrast, when LAA approaches 0–20° or 80–90°, the corresponding SHAP values decrease overall, suggesting that twinning becomes less favorable when the overall grain elongation direction is either nearly parallel or nearly perpendicular to the tensile direction. These results indicate that an intermediate angle between the grain long-axis direction and the external loading direction is more favorable for twinning occurrence.

From a physical perspective, LAA should not be interpreted as a parameter that directly characterizes the mechanical properties of an individual grain boundary. Rather, it is more appropriately regarded as a statistical descriptor of the overall geometric elongation direction of a grain. For real irregular grains, the outer contour is composed of numerous local grain-boundary segments. As the grain long axis changes its orientation relative to the tensile direction, the orientation distribution and connectivity of these local boundary segments also change in a statistical manner. Based on these results, we hypothesize that the influence of LAA on twinning does not arise from a direct modification of the properties of a specific grain boundary. Instead, it may be associated with changes in the combinations of boundary-segment orientations, which could further affect the local constraint state, stress-transfer paths, and effective boundary resistance around the grain. In other words, we propose that LAA reflects not only the direction in which a grain is elongated, but also the differences in boundary-orientation combinations associated with the overall grain-shape orientation, and that these differences may modulate local stress concentration and deformation compatibility conditions, thereby influencing the probability of twinning occurrence. It should be emphasized that SHAP identifies statistical associations between features and model predictions, rather than causal relationships. The boundary-orientation interpretation proposed above is therefore a hypothesis derived from these statistical trends, and its direct validation requires further dedicated investigation.

### 3.4. Representative EBSD Observations Consistent with the Framework-Derived Insights

The previous section demonstrated, through SHAP analysis of all 1011 grains, that LAA exhibits a population-level statistical trend characterized by promotion in the intermediate range and suppression at both ends. Specifically, grains with LAA values within 20–80° showed a higher twinning propensity, whereas twinning probability decreased when LAA approached 0–20° or 80–90°. While this statistical finding constitutes the primary evidence for the LAA effect, this section presents representative grain-level observations from in situ EBSD to provide a visual illustration of the physical meaning of LAA and to examine whether these individual cases are consistent with the population-level statistical trend. The discussion focuses on how the overall geometric elongation direction of a grain may alter the combination of boundary-segment orientations, thereby influencing local constraint conditions, stress-transfer paths, and effective boundary resistance, and ultimately modulating twinning behavior.

**Figure 12:**
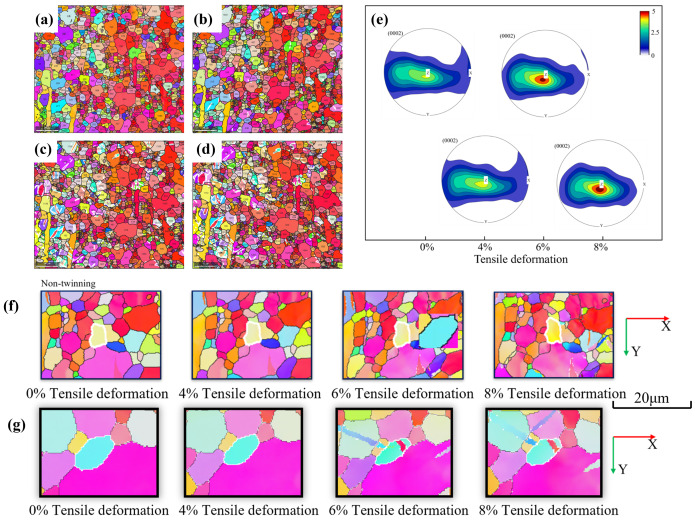
Analysis of the specific grain during the deformation process. (**a**–**d**) IPF maps of the observed region at strains of 0%, 4%, 6%, and 8%. (**e**) Pole figures showing texture evolution of the observed region at the corresponding strains. (**f**) IPF deformation process map for a representative grain predicted as non-twinned (confirmed by EBSD). (**g**) IPF deformation process map for a representative grain predicted as twinned (confirmed by EBSD).

Figure 12a–d show the microstructural evolution of the observed region during deformation, and Figure 12e presents the corresponding pole figures illustrating texture evolution. Figure 12f,g further present two representative grain-level examples. The two grains have relatively similar values of key features that strongly affect twinning behavior, such as Diameter and SFTensionTwin3 (SF3), but they differ markedly in LAA. Therefore, they can be used as representative cases to discuss the possible relationship between grain-shape orientation and twinning response.

As shown in Figure 12f, the representative grain has an LAA close to 90°, and a relatively large fraction of its boundary segments are oriented nearly parallel or perpendicular to the tensile direction. Although this grain underwent appreciable plastic deformation and was affected by deformation transfer from neighboring grains during loading, no new twin nucleation was observed within the grain throughout the deformation process. This observation suggests that, under similar grain-size conditions and twinning Schmid factors, when the boundary-segment combination associated with the overall grain elongation direction is dominated by segments nearly parallel or perpendicular to the tensile direction, it may be less favorable for generating local stress concentration and effective stress-transfer paths that promote twinning. Consequently, even though this grain experienced noticeable plastic deformation, it remained untwinned.

In contrast, the grain shown in Figure 12g has an LAA of approximately 40°, and many of its boundary segments are distributed within the 20–80° range, corresponding to a more typical inclined-boundary configuration. This grain eventually formed a clear twin, which nucleated near one inclined grain boundary and then propagated into the grain interior in a band-like morphology until it reached the opposite boundary. This observation is consistent with the SHAP-derived statistical trend that LAA values within the 20–80° range promote twinning. It also suggests that, when the overall geometric elongation direction of a grain forms a moderate angle with the tensile direction, the corresponding boundary-segment orientation combination may be more favorable for local stress transfer and the accumulation of accommodation mismatch, thereby providing more favorable structural conditions for twin nucleation and subsequent propagation.

### 3.5. MD-Based Mechanical Support for the Boundary-Segment Interpretation

To provide mechanical support for the boundary-segment orientation interpretation of the LAA effect identified by SHAP analysis, molecular dynamics (MD) simulations were performed. It is important to note that these MD simulations employ simplified bicrystal geometries that do not capture realistic grain morphology or the full twinning process. Nevertheless, they allow the mechanical responses of local boundary segments with different orientations relative to the tensile direction to be isolated, providing indirect evidence for the mechanical plausibility of the proposed boundary-segment interpretation.

**Figure 13:**
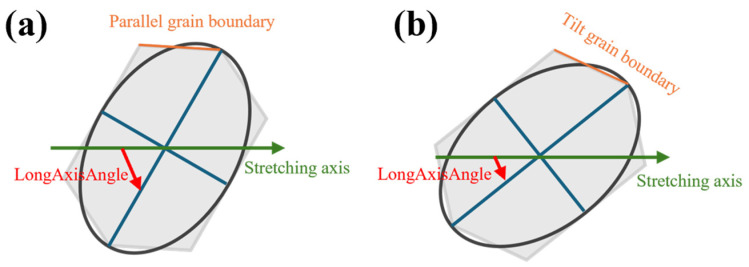
Schematic illustration of the relationship between the fitted elliptical long-axis angle and the orientation distribution of local grain-boundary segments relative to the tensile direction. (**a**) Relationship between grain boundaries and tensile direction before rotation; (**b**) Relationship between grain boundaries and tensile direction after rotation.

To clarify the connection between LAA and local boundary-segment orientation, an idealized schematic grain is shown in Figure 13. For an elongated grain, the fitted elliptical long axis provides a statistical description of the overall grain-shape orientation. Because the boundary of an irregular grain can be regarded as being composed of many short boundary segments, changing the long-axis angle relative to the tensile direction also changes the statistical distribution of these boundary-segment orientations. Therefore, LAA should not be interpreted as a direct mechanical property of a single boundary, but as a descriptor reflecting the boundary-segment orientation combinations associated with grain elongation.

**Figure 14:**
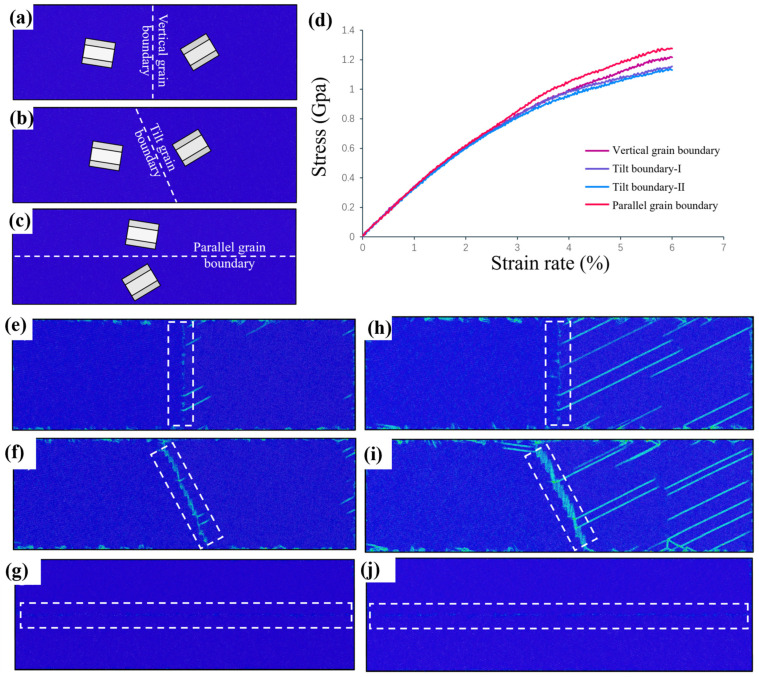
MD simulations of tensile models: (**a**–**c**) three models with different grain-boundary orientations; (**d**) stress–strain curves of the three models; (**e**–**j**) atomic strain maps at the middle tensile stage (**e**–**g**); and final tensile stage (**h**–**j**).

Based on this interpretation, simplified MD tensile models with different boundary-segment orientations were constructed, as shown in Figure 14a–c. The stress–strain curves in Figure 14d indicate that boundary segments oriented nearly parallel or perpendicular to the tensile direction, corresponding approximately to 0–20° or 80–90°, exhibit higher resistance to deformation. In contrast, inclined boundary segments within the 20–80° range show lower resistance and greater deformation accommodation.

To exclude the possibility that this behavior originates from a specific left–right arrangement of the model, an alternative inclined-boundary model was constructed by swapping the left and right orientations of the model in Figure 14b. The resulting mechanical response is similar to that of the original inclined-boundary model, with only minor differences, indicating that the observed response is mainly associated with boundary-segment orientation rather than a specific geometric arrangement.

Atomic strain maps in Figure 14e–j further show that parallel or perpendicular boundary segments undergo relatively limited deformation, whereas inclined boundary segments exhibit more pronounced local strain accumulation. These results suggest that inclined boundary segments can provide more favorable deformation accommodation and stress-transfer conditions, which are relevant to twin nucleation.

## 4. Conclusions

This study established an Optuna–SHAP interpretable machine-learning framework for predicting twinning nucleation in Mg alloys. The main conclusions are as follows:

The integrated Optuna–SHAP approach improves the predictive robustness of different machine-learning models while retaining interpretability. SHAP analysis reproduced known feature–twinning relationships in public datasets, indicating that the optimized models capture physically consistent trends in the examined systems.

In the self-constructed in situ EBSD dataset, the long-axis angle (LAA) was identified as a secondary but independent grain shape-orientation descriptor. Although LAA is not the dominant factor controlling twinning nucleation, it provides additional information beyond conventional grain-size and projected-size descriptors in the examined alloy and deformation conditions.

The LAA effect exhibits an orientation-dependent trend: grains with long-axis orientations of approximately 20–80° relative to the tensile direction are more frequently associated with twinning nucleation, whereas orientations close to 0–20° or 80–90° tend to show lower twinning probability in the AZ31 alloy system studied. EBSD observations and MD simulations are consistent with a boundary-segment-based hypothesis for this behavior, suggesting that changes in boundary-segment orientation combinations may regulate local constraint, stress transfer, deformation accommodation, and effective boundary resistance. Further work is needed to directly validate this proposed mechanism.

## Data Availability

The original contributions presented in this study are included in the article. Further inquiries can be directed to the corresponding author.
